# Pharmacological strategies for myocardial protection in cardiac surgery: current science and the emerging role of multi-omics and artificial intelligence

**DOI:** 10.3389/fcvm.2026.1844417

**Published:** 2026-09-04

**Authors:** Hanad Ahmed, Jason Ali

**Affiliations:** Department of Cardiothoracic Surgery, Royal Papworth Hospital, Cambridge, United Kingdom

**Keywords:** artificial intelligence, biomarkers, cardiac surgery, cardioplegia, cardiopulmonary bypass, coronary artery bypass grafting, cyclosporin A, endothelial glycocalyx

## Abstract

Perioperative myocardial ischaemia-reperfusion injury continues to represent a significant source of morbidity and mortality in patients undergoing cardiac surgery, despite considerable advances in cardiopulmonary bypass technology and operative technique. Contemporary cardioprotective approaches, including cardioplegia, offer only partial attenuation of this injury. Intraoperative protection strategies have evolved little over recent decades, and clinical trials investigating pharmacological agents directed at ischaemia-reperfusion pathways have yielded inconsistent results, though subgroup analyses frequently reveal benefit in specific patient populations. Such variability may reflect the inherent limitations of applying standardised interventions to physiologically diverse patient cohorts. Multi-omics profiling offers the capacity to identify distinct molecular signatures associated with susceptibility to ischaemia-reperfusion injury and severity of myocardial damage, and when combined with artificial intelligence, may predict individual therapeutic responses. These bioinformatic advances have implications not only for risk stratification and treatment selection but also for the design of future clinical trials through improved patient selection. This review synthesises current evidence regarding the mechanisms of ischaemia-reperfusion injury and evaluates pharmacological interventions targeting mitochondrial dysfunction, microvascular impairment, and inflammatory pathways. The potential role of multi-omics technologies integrated with artificial intelligence in enabling precision approaches to myocardial protection is subsequently examined.

## Introduction

Despite advances in cardiopulmonary bypass technology and surgical technique, patients undergoing cardiac surgery continue to sustain clinically meaningful myocardial injury, which manifests as elevated cardiac biomarkers, impaired ventricular function, arrhythmias, and in severe instances, low cardiac output syndrome and death (1, 2). The increasing age and comorbidity burden of the surgical population has compounded this challenge, as more complex pathology necessitates longer and more technically demanding procedures with extended aortic cross-clamp and ischaemic times. Consequently, myocardial protection has become a critical focus of research in cardiac surgery.

Myocardial injury arises through multiple mechanisms, encompassing direct surgical trauma, inflammation, oxidative damage, calcium dysregulation, mitochondrial dysfunction, and microvascular impairment, each capable of compromising contractile performance (2). The ischaemia-reperfusion injury (IRI) phenomenon is central to many of these pathways (2). The ischaemic phase commences with cardioplegic arrest and aortic cross-clamping, during which cessation of coronary perfusion initiates metabolic derangement (3). Cellular energy generation shifts from efficient aerobic pathways to less effective anaerobic metabolism, resulting in rapid depletion of adenosine triphosphate (ATP) and accumulation of metabolic byproducts including lactate, phosphate compounds, and hydrogen ions (3, 4). These alterations impair cellular ion transport, disrupt calcium homeostasis, and promote myocardial oedema (4). Upon removal of the aortic cross-clamp and restoration of coronary perfusion, a paradoxical reperfusion injury compounds the preceeding ischaemic damage. Reintroduction of oxygen generates reactive oxygen species (ROS) that overwhelm endogenous antioxidant defences. The resulting oxidative stress damages membrane lipids, thereby compromising membrane integrity and ion channel function (4). Oxidation of cellular enzymes further impairs function, whilst direct DNA damage activates programmed cell death pathways, culminating in cardiomyocyte loss. This destructive sequence unfolds rapidly following restoration of coronary flow (4, 5).

Cardioplegia constitutes the cornerstone of myocardial protection during cardiac surgery. Cardioplegic solutions are broadly categorised as extracellular or intracellular formulations, each achieving diastolic arrest through distinct mechanisms. Extracellular solutions, including St Thomas' and Del Nido formulations, are hyperkalaemic preparations in which raised extracellular potassium depolarises the resting membrane potential, inactivating fast sodium channels and arresting the heart in depolarised diastolic standstill (5). Intracellular solutions, most notably histidine-tryptophan-ketoglutarate (HTK/Custodiol), are characterised by low sodium and calcium concentrations and arrest the heart by depleting the transmembrane sodium gradient such that the threshold for action potential generation cannot be reached, maintaining the myocyte in a polarised quiescent state near its resting membrane potential (6). Administration methods vary according to temperature and composition, whilst delivery routes include antegrade administration through the coronary ostia or retrograde delivery via the coronary sinus.

Notable distinctions between the two most commonly employed extracellular formulations include Del Nido's incorporation of lidocaine, mannitol, and magnesium sulphate, together with reduced calcium concentrations compared with St Thomas’ solution. This is thought to enhance membrane stabilisation and attenuate oxidative stress (7). Whilst cardioplegia provides the foundation for myocardial protection, it does not address specific ischaemia-reperfusion mechanisms to mitigate perioperative myocardial injury. This uniform approach fails to account for the substantial heterogeneity among cardiac surgical patients that underpins their response to ischaemic stress and reperfusion injury.

Recent scientific and technological developments have enabled exploration of more individualised approaches to myocardial protection and broader cardiac surgical care. The emergence of omics technologies has facilitated a shift towards personalised molecular profiling to inform clinical management (8). Multi-omics integrates patient genomics, transcriptomics, proteomics, and metabolomics, constructing a comprehensive view of the molecular determinants of disease responses (8). When combined with artificial intelligence and machine learning, this multimodal biological data offers unprecedented opportunities for personalised risk stratification and tailored therapeutic strategies (9). This review examines the pathophysiology of IRI and its clinical implications before exploring the mechanistic basis of emerging pharmacological therapies for myocardial protection. Subsequently, it will assess the emerging role of multi-omics technologies and artificial intelligence in advancing personalised myocardial protection in cardiac surgery.

Numerous reviews have characterised the mechanisms of myocardial ischaemia-reperfusion injury, the principles of cardioplegic arrest, and the pharmacology of individual cardioprotective agents. These have typically treated the persistent failure of such agents in clinical trials as a problem of insufficient drug efficacy or suboptimal trial design. The present review is distinct in three respects. First, it reframes the inconsistent trial record not as evidence that cardioprotective pharmacology has failed, but as the predictable consequence of applying uniform interventions to a molecularly heterogeneous surgical population. Second, rather than cataloguing agents in isolation, it explicitly maps emerging pharmacological therapies onto the specific pathophysiological mechanisms they target, namely mitochondrial dysfunction, microvascular impairment, and systemic inflammation, and onto the molecular phenotypes most likely to respond to each. Third, it integrates this mechanistic synthesis with a forward-looking framework in which multi-omics profiling and artificial intelligence are used to identify those phenotypes prospectively and to match each patient to a mechanism-specific cardioprotective strategy. In doing so, the review connects two literatures that are usually considered separately, cardioprotective pharmacology and precision cardiovascular medicine, and proposes a practical, perioperatively grounded pathway towards individualised myocardial protection.

## Methods

This article is a narrative review. A structured but non-systematic literature search was undertaken to identify evidence across three domains: pharmacological agents for myocardial protection, multi-omics profiling relevant to ischaemia-reperfusion injury, and artificial intelligence applied to cardiovascular risk prediction and molecular data integration. MEDLINE (via PubMed), Embase, the Cochrane Central Register of Controlled Trials, and Web of Science were searched, with Google Scholar and citation chaining (backward and forward snowballing of key references) used to identify additional sources. Searches combined controlled vocabulary and free-text terms relating to the core concepts, for example “ischaemia-reperfusion injury”, “myocardial protection”, “cardioplegia”, “cardiopulmonary bypass”, and “cardiac surgery”, paired with agent-specific terms (“cyclosporin A”, “elamipretide”, “coenzyme Q10”, “mitoquinol”, “colchicine”, “N-acetylcysteine”, “corticosteroids”, “doxycycline”, “dexmedetomidine”) and with methodological terms (“genomics”, “epigenomics”, “transcriptomics”, “proteomics”, “metabolomics”, “multi-omics”, “machine learning”, “artificial intelligence”, “network medicine”). Foundational mechanistic and conceptual papers were included irrespective of publication date; for clinical, multi-omics, and artificial intelligence evidence, emphasis was placed on literature published between 2005 and 2026, reflecting the relative recency of these fields. Only English-language publications were considered.

Inclusion logic was applied separately to each domain. For pharmacological agents, randomised controlled trials and meta-analyses in cardiac surgical populations were prioritised; preclinical and mechanistic studies were included where human cardiac surgical data were limited but the agent had a defined molecular target relevant to mitochondrial, microvascular, or inflammatory injury. For multi-omics studies, those applying genomic, epigenomic, transcriptomic, proteomic, or metabolomic profiling to cardiac surgical or ischaemia-reperfusion-relevant cohorts were included, with broader cardiovascular omics literature drawn upon where cardiac-surgery-specific data were sparse. For artificial intelligence, studies applying machine learning to cardiovascular outcome prediction, multi-omics integration, or network-medicine approaches were included, alongside methodological and ethical literature addressing model interpretability, generalisability, and algorithmic bias. Given the narrative design, final selection reflected the authors’ assessment of mechanistic relevance, methodological quality, and contribution to a coherent synthesis rather than a predefined systematic protocol.

### Pathophysiology of ischaemia-reperfusion injury and the redox paradox

**Figure 1:**
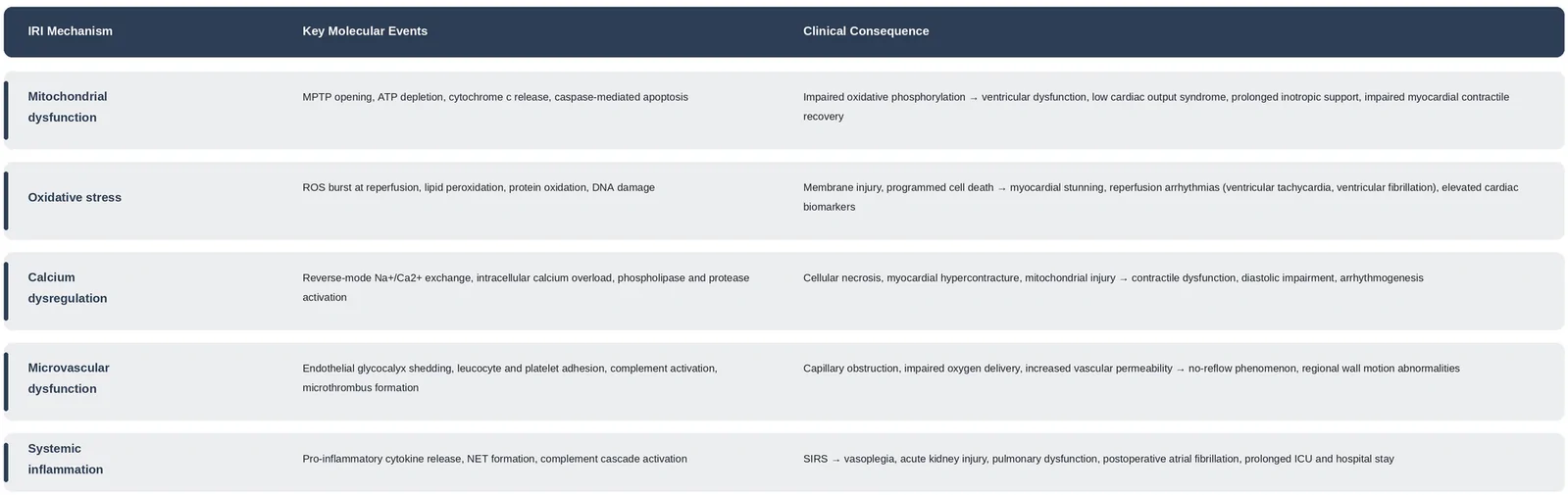
Principal mechanisms of ischaemia-reperfusion injury in cardiac surgery [adapted from (2, 4, 5, 14)].

IRI is not a single event but a coordinated cascade of overlapping pathological processes initiated at the onset of myocardial ischaemia and amplified upon reperfusion (2). As outlined as outlined previously, the ischaemic phase is characterised by metabolic failure, ionic dysregulation, and calcium overload, whilst reperfusion compounds this through oxidative stress, opening of the mitochondrial permeability transition pore (MPTP) opening, and microvascular injury. Critically, these mechanisms are not only independently damaging but mutually reinforcing, amplifying cardiomyocyte loss and ventricular dysfunction in ways that a single cardioprotective strategy is unlikely to fully address. The principal mechanisms of IRI and their clinical consequences are summarised in Figure 1. Each of these mechanisms is considered in detail alongside the agents that target them in the sections that follow. Recent network-pharmacology analyses similarly map these interdependent processes, oxidative stress, calcium overload, mitochondrial dysfunction, and inflammation, onto shared and interacting molecular targets, reinforcing the view that effective cardioprotection is unlikely to be achieved by addressing any single pathway in isolation (10).

This emphasis on the damaging consequences of reactive oxygen species should not, however, obscure their dual role in cardiomyocyte biology. Low-level, transient ROS production, particularly from mitochondrial sources during brief sublethal ischaemic episodes, functions as a signalling event that activates the reperfusion injury salvage kinase (RISK) and survivor activating factor enhancement (SAFE) pathways and mediates the cardioprotective benefits of ischaemic preconditioning and postconditioning (11). Whether ROS act as protective signalling molecules or as damaging effectors depends on their source, concentration, subcellular compartment, and the timing of their generation relative to ischaemia.

This redox duality has important translational implications: indiscriminate antioxidant strategies, by suppressing both pathological and protective ROS, may attenuate not only injury but also endogenous cardioprotective signalling, providing one plausible mechanistic explanation for the disappointing performance of broad antioxidants such as N-acetylcysteine in cardiac surgical trials despite favourable biochemical effects. The timing of antioxidant administration relative to ischaemia and reperfusion is itself likely to be a decisive determinant of efficacy: agents delivered before or during the ischaemic conditioning window risk abolishing the protective ROS signalling that mediates preconditioning and postconditioning, whereas benefit may be confined to interventions targeted specifically at the injurious oxidative burst that accompanies reperfusion (12, 13).

### Clinical relevance of ischaemia-reperfusion injury

IRI manifests clinically through several important pathways in cardiac surgery. Perioperative myocardial infarction occurs in 0.3%–9.8% of patients undergoing Coronary Artery Bypass Grafting (CABG), contributing to increased mortality and long-term heart failure risk (15). Myocardial stunning, defined as prolonged contractile dysfunction despite restored coronary flow, extends inotropic support requirements and ICU length of stay (16). Reperfusion arrhythmias, particularly AF, affect approximately 32% of patients after CABG (17, 18). In severe cases, IRI precipitates low cardiac output syndrome necessitating mechanical circulatory support.

Substantial heterogeneity exists in the clinical manifestations of IRI despite similar operative characteristics, with individual cases ranging from subclinical biomarker elevation to low cardiac output syndrome (19). Current cardioplegia-based strategies fail to account for this variability, applying identical protection to patients with vastly different baseline risk profiles, ischaemic tolerance, and injury susceptibility. Addressing this gap requires omics-informed risk stratification to identify high-risk individuals and mechanism-specific pharmacological strategies tailored to individual biology. The following sections examine emerging pharmacological interventions targeting these mechanisms, before considering how multi-omics and artificial intelligence may enable truly individualised myocardial protection.

### Mechanistic targets and emerging therapies

The agents discussed in this section were selected to illustrate the three principal, mechanistically distinct targets of ischaemia-reperfusion injury rather than to provide an exhaustive pharmacopoeia. Cyclosporin A, elamipretide, and mitoquinol/coenzyme Q10 were chosen as representative agents acting on mitochondrial dysfunction, addressing permeability transition pore opening, cardiolipin-dependent cristae integrity, and reperfusion oxidative stress respectively. Doxycycline and dexmedetomidine were selected as agents targeting microvascular dysfunction, through inhibition of matrix metalloproteinase-mediated glycocalyx degradation and attenuation of endothelial inflammation.

Colchicine, N-acetylcysteine, and corticosteroids were included as agents addressing the systemic inflammatory component of injury. Selection prioritised agents with a defined molecular mechanism relevant to one of these pathways together with at least preliminary translational or clinical data in cardiac surgery or a closely related cardiovascular setting; agents of mainly historical interest, or those without a plausible route to perioperative use, were not considered.

#### Mitochondrial dysfunction in ischaemia-reperfusion injury

Mitochondrial impairment occupies a central position in IRI. During ischaemia, reduced adenosine triphosphate production disrupts ion pumps including the sodium-potassium exchanger and calcium efflux channels, causing ionic imbalances and cellular swelling (4). Upon reperfusion, sodium-calcium exchange mechanisms exacerbate cytosolic calcium overload (4, 5). Mitochondria attempt to buffer excess calcium, but overload triggers sustained opening of the mitochondrial permeability transition pore (MPTP) (5). This causes mitochondrial swelling, membrane rupture, and collapse of the electrochemical gradient, halting ATP production (5). Release of pro-apoptotic factors including cytochrome c activates caspase-mediated apoptosis, and damaged mitochondria trigger an immune response. These processes ultimately cause cardiomyocyte death and cardiac dysfunction (4, 5).

#### Experimental therapies targeting mitochondrial dysfunction

##### Cyclosporin A (CsA)

The central role of mitochondrial dysfunction in IRI has established it as a priority therapeutic target. Most interventions have focused on providing membrane and structural stability to prevent mitochondrial dysfunction, rupture, and subsequent activation of apoptotic pathways.

Cyclosporin A is a recognised inhibitor of the mitochondrial permeability transition pore and has been investigated for its potential to reduce IRI in cardiac surgery. Clinical evidence presents mixed results, with outcomes appearing sensitive to timing of administration, surgical procedure type, and degree of ischaemic burden. In elective aortic valve surgery, Chiari and colleagues conducted a single-centre randomised trial involving 61 patients in which 2.5 mg/kg of intravenous cyclosporin A was administered approximately 10 min before aortic cross-clamp removal (20). This resulted in an approximately 35% reduction in 72-h troponin-I area under the curve compared with placebo, although no clear differences in clinical outcomes were observed. In the context of elective CABG, Hausenloy and colleagues reported no overall reduction in perioperative myocardial injury with a single intravenous bolus administered prior to surgery; however, *post hoc* analysis indicated potential benefit in patients experiencing longer CPB times, suggesting that cyclosporin A efficacy may depend on ischaemic load (21, 22). In terms of evidence level, cyclosporin A is supported by direct but small randomised controlled trials in cardiac surgery, with results that remain inconsistent across procedures.

##### Elamipretide

Elamipretide, an aromatic-cationic peptide, represents a promising therapeutic approach in animal models of cardiac IRI and in clinical studies of heart failure and renal artery stenosis. It selectively binds to cardiolipin in the inner mitochondrial membrane and has demonstrated potential to mitigate disintegration of the mitochondrial cristae network in experimental models of IRI (23). Cardiolipin, a unique phospholipid with four fatty acid side chains situated in the inner mitochondrial membrane, plays a vital role in maintaining the structural integrity and function of the mitochondrial respiratory chain complexes (24). During stress, the natural ageing process, and pathological conditions including IRI and heart failure, cardiolipin content diminishes, altering its interaction with respiratory chain proteins, particularly cytochrome c (24).

Whilst elamipretide does not prevent the reduction of cardiolipin content under such conditions, animal studies suggest it may help stabilise the cardiolipin-cytochrome c interaction, supporting mitochondrial membrane integrity and improving supercomplex coupling (23, 25). Several studies have evaluated the efficacy of elamipretide in improving cardiac and renal function with promising results (26, 27).

In explanted failing human hearts from both paediatric and adult patients, elamipretide treatment rapidly improved mitochondrial oxygen flux, enhanced activities of complexes I and IV, and increased supercomplex-associated complex IV activity, independent of cardiolipin remodelling (28). In a first-in-human phase 2 trial, a single 4-h infusion of high-dose elamipretide in patients with heart failure with reduced ejection fraction demonstrated excellent safety and tolerability whilst producing significant reductions in left ventricular end-diastolic and end-systolic volumes that correlated with peak plasma concentrations (27).

However, a subsequent phase 2 trial randomising 71 patients with heart failure with reduced ejection fraction to 28 days of subcutaneous elamipretide found no improvement in left ventricular end-systolic volume, end-diastolic volume, or ejection fraction, indicating that the structural benefit observed after a single infusion was not sustained with repeat dosing (107). Although large cardiac surgical trials evaluating elamipretide efficacy in the perioperative setting have yet to be established, this evidence from heart failure studies demonstrates safety and tolerability and suggests potential for mitochondrially targeted therapies in reducing cardiac IRI. It should be emphasised that this evidence is preclinical and indirect: the cardiac data derive from animal ischaemia-reperfusion models and from heart failure trials, and no randomised trial has yet evaluated elamipretide in cardiac surgery, so its application to perioperative myocardial protection currently remains theoretical.

##### Coenzyme Q10 and mitochondrially targeted derivatives

Coenzyme Q10 is an endogenous electron carrier of the respiratory chain with antioxidant activity at the inner mitochondrial membrane, and it has been pursued for myocardial protection in two forms: as oral supplementation, and as a mitochondrially targeted derivative designed to overcome the poor mitochondrial accumulation that limits the oral compound.

In cardiac surgical patients, several small randomised trials have evaluated preoperative coenzyme Q10 supplementation. Rosenfeldt and colleagues demonstrated that coenzyme Q10 at 300 mg per day for two weeks increased myocardial coenzyme Q10 concentrations, improved mitochondrial efficiency, and enhanced *in vitro* contractile recovery after hypoxia-reoxygenation, though no significant differences in postoperative haemodynamics or troponin release were observed (34).

**Figure 2:**
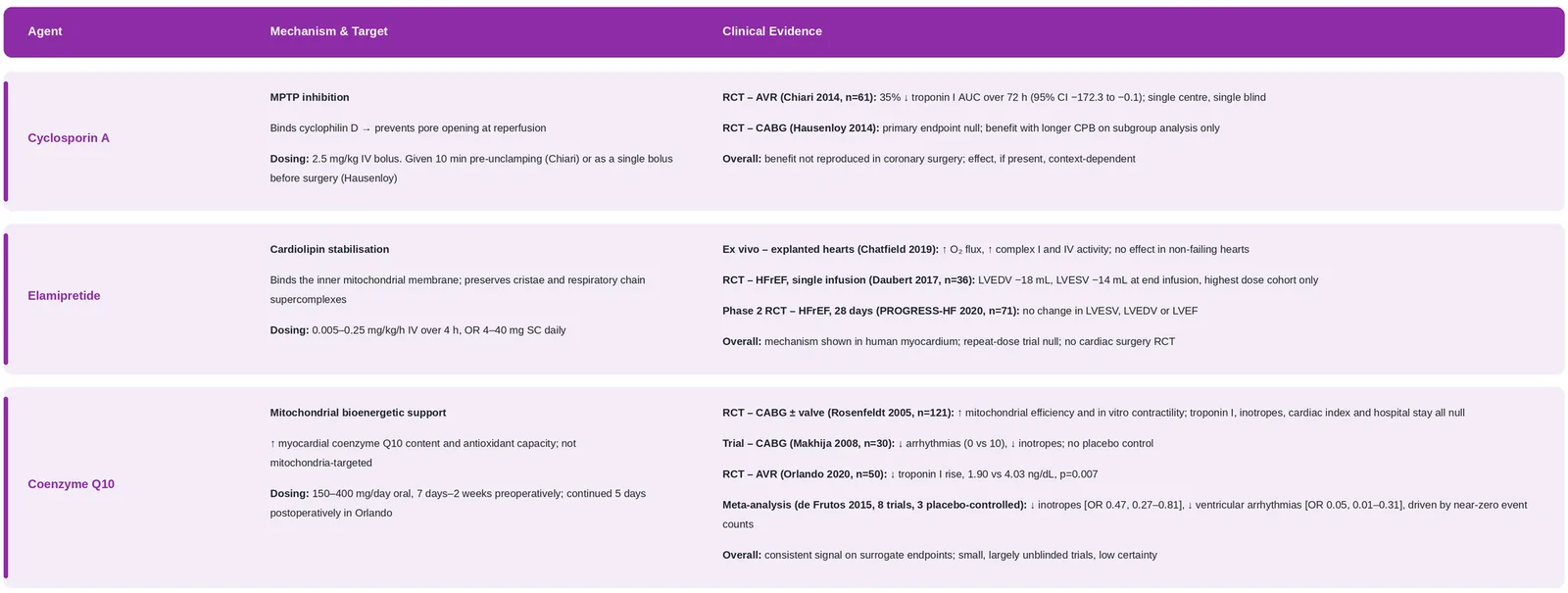
Mitochondrial-targeted therapeutic strategies for ischaemia-reperfusion injury.

Subsequent trials reported clinical benefits: Makhija and colleagues found reduced reperfusion arrhythmias, lower inotropic requirements, and shorter hospital stays in CABG patients (35), whilst Orlando and colleagues demonstrated reduced troponin release and improved left ventricular ejection fraction recovery in elderly patients undergoing aortic valve replacement (36). A meta-analysis of eight trials confirmed that prophylactic coenzyme Q10 significantly reduces postoperative inotropic requirements and ventricular arrhythmias (37). These findings suggest that raising myocardial coenzyme Q10 availability may confer perioperative benefit, although the null troponin and haemodynamic results of the earliest trial temper that conclusion and larger studies are needed to establish definitive recommendations. Oral supplementation is also constrained by limited delivery to the mitochondrial compartment where the protective effect is required. Mitoquinone mesylate (MitoQ) was developed to address this limitation. It is a coenzyme Q10 derivative conjugated to a triphenylphosphonium cation, which drives its accumulation within mitochondria and allows it to target the oxidative stress that underpins mitochondrial damage during reperfusion (29, 30). Administration prior to ischaemia in animal models of IRI reduces injury by decreasing mitochondrial swelling, cytochrome c release and subsequent cell death (31–33). The requirement for pretreatment, which constrains translation in most ischaemic syndromes, is well suited to elective cardiac surgery, where the ischaemic insult is scheduled and preoperative loading is straightforward. MitoQ has not, however, been evaluated in cardiac surgery, and the trial data above relate to oral coenzyme Q10, a mechanistically related but pharmacologically distinct compound. The two should not be regarded as interchangeable, and the absence of cardiac surgical data for MitoQ represents a tractable opportunity for further research. These mitochondrially targeted strategies and their current evidence base are summarised in Figure 2.

#### Microvascular dysfunction in ischaemia-reperfusion injury

Microvascular impairment following IRI can persist despite successful coronary flow restoration, a phenomenon termed no-reflow (14). Enhanced capillary permeability causes cellular swelling, reducing microvascular diameter and promoting coagulation activation with microthrombus formation (14, 38). Microcirculatory obstruction from cellular debris, microthrombi, and endothelial dysfunction collectively impairs tissue perfusion despite restored epicardial coronary flow (14, 38). Although this phenomenon is more widely studied in the context of percutaneous coronary intervention, it represents a critical mechanism of IRI that may occur during cardiac surgery. Several studies have demonstrated microcirculatory dysfunction at CPB onset, which can persist for up to 72 h (39, 40). The extent of injury correlates with CPB duration (39). The thesis of microvascular dysfunction occurring as a result of global IRI in cardiac surgery is further supported by observations that microcirculatory perfusion is preserved during off-pump surgery compared with on-pump surgery (41), suggesting that IRI leads to microvascular dysfunction.

Critically, these changes occur in the context of normal physiological parameters including mean arterial pressure and blood gases, highlighting the disconnect between macrovascular and microvascular perfusion (39). A key amplifier of no-reflow is endothelial glycocalyx shedding, which is well described during CPB and early reperfusion (42, 43).

Loss of the endothelial glycocalyx exposes adhesion molecules, reduces nitric oxide bioavailability, increases permeability, and promotes leucocyte and platelet plugging (42). Common comorbidities including hypertension, ageing, obesity, and particularly diabetes prime this process via ROS and advanced glycation end-products, rendering some patients intrinsically more vulnerable (44).

Beyond endothelial and glycocalyx pathology, capillary-level regulatory mechanisms make a substantial and increasingly recognised contribution to no-reflow. Pericytes, contractile mural cells that wrap around capillaries, undergo ischaemia-induced contraction driven by reactive oxygen and nitrogen species and by cytosolic calcium accumulation, narrowing capillary lumens at discrete constriction points; these constrictions can persist despite restoration of upstream coronary flow, mechanically obstructing red cell passage even in patent vessels (45, 46). O'Farrell and colleagues extended this paradigm directly to the heart, demonstrating in a rat coronary occlusion-reperfusion model that approximately 40% of cardiac capillaries are blocked and perfused volume halved in the affected region, with capillary blockages co-localising with pericyte somata where capillary diameter was reduced by 37%; the pericyte relaxant adenosine partially reversed this constriction and substantially restored perfusion (47). These observations indicate that pericyte-mediated capillary constriction operates in concert with endothelial and glycocalyx pathology and help to explain why epicardial coronary patency may be a poor surrogate for genuine tissue-level perfusion. Therapeutic strategies that preserve endothelial integrity without also addressing pericyte function are therefore unlikely to fully resolve microvascular dysfunction in cardiac surgery.

#### Experimental strategies targeting microvascular dysfunction

Several approaches exist to target microvascular dysfunction, and the most effective strategy is multimodal and mechanism-informed. Preservation and, where feasible, reconstitution of the endothelial glycocalyx represents a key therapeutic target. This includes minimising CPB duration, avoiding haemodilution, reducing shear and air-blood exposure, and using pharmacological agents to limit enzymatic breakdown of glycocalyx components (39, 42, 48, 49). Pharmacological protection of the endothelial glycocalyx during cardiac surgery encompasses both inhibition of enzymatic degradation and activation of pro-repair signalling pathways. Enzymatic breakdown involves multiple proteases, particularly matrix metalloproteinases, that degrade glycocalyx components, reducing protective barrier function and increasing endothelial permeability and leucocyte adhesion (48). Recent trials in cardiac surgery have demonstrated doxycycline and dexmedetomidine as potential therapeutic interventions to reduce glycocalyx shedding, by inhibiting matrix metalloproteinases and reducing inflammation respectively, with promising results (49, 50). The clinical evidence for doxycycline and dexmedetomidine in this context is, however, currently limited to single small randomised trials reporting surrogate markers of glycocalyx integrity rather than patient-centred clinical outcomes.

**Figure 3:**
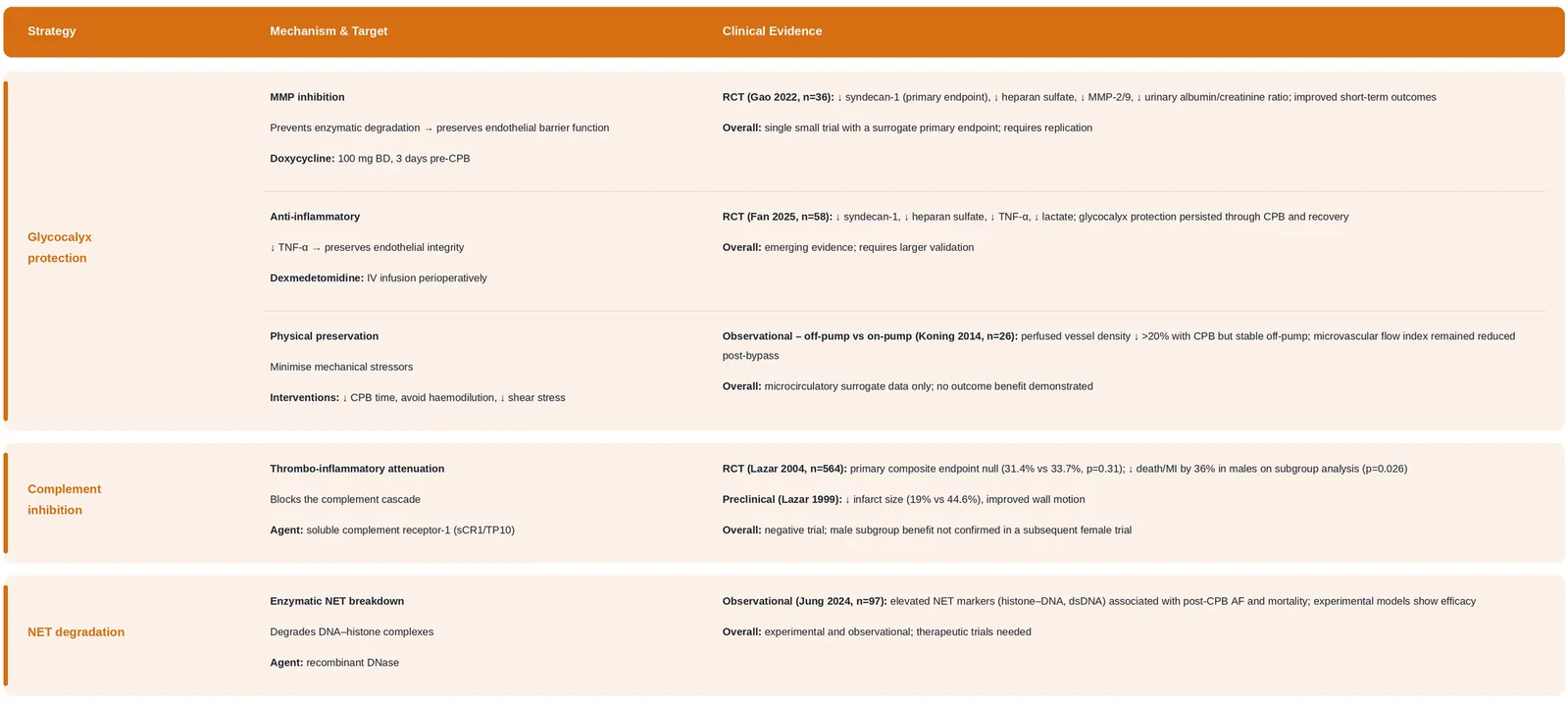
Microvascular dysfunction therapeutic strategies.

The thrombo-inflammatory component of microvascular dysfunction represents another key area for therapeutic intervention and is characterised by complement activation, neutrophil infiltration, platelet aggregation, and microvascular thrombosis. Several clinical and preclinical studies have demonstrated the therapeutic potential of complement inhibition for attenuating microvascular injury after cardiac surgery, with a 36% reduction in death or myocardial infarction observed in male patients, although the trial was neutral for its primary composite endpoint and the effect was not confirmed in a subsequent trial in female patients (51–53). Similarly, neutrophil extracellular traps constitute a critical inflammatory mediator, with elevated circulating levels observed during CPB correlating with adverse outcomes (54). Enzymatic degradation of these DNA-histone complexes using recombinant DNase has shown efficacy in reducing endothelial activation, limiting leucocyte extravasation, and improving microvascular function in experimental cardiac surgery models of IRI (55). The strategies targeting microvascular dysfunction, and the level of evidence supporting each, are summarised in Figure 3.

#### Anti-inflammatory strategies

Anti-inflammatory strategies provide potential benefit across all aspects of the IRI response and can ameliorate the release of pro-inflammatory mediators that lead to mitochondrial and microvascular impairment. They have therefore become a central therapeutic target.

##### Colchicine

Colchicine has been evaluated in several cardiac surgery trials. Pan and colleagues conducted a randomised controlled trial in patients undergoing non-coronary artery bypass cardiac surgery, demonstrating that low-dose colchicine at 0.5 mg twice daily for three days perioperatively significantly reduced postoperative myocardial injury markers and systemic inflammatory cytokines whilst maintaining a favourable safety profile (56). The COPPS trial established that colchicine 1.0 mg administered within three days of cardiac surgery, followed by 0.5 mg daily for one month, significantly reduced postoperative atrial fibrillation rates (57). However, the subsequent COPPS-2 trial did not demonstrate a statistically significant reduction in atrial fibrillation in the intention-to-treat analysis, though it did significantly reduce postpericardiotomy syndrome (58). Meta-analyses of multiple trials support overall efficacy for atrial fibrillation reduction, though gastrointestinal adverse events are increased (59, 60). Despite these findings, several questions remain regarding optimal dosing strategies, timing of initiation relative to surgical intervention, treatment duration, and management of gastrointestinal adverse events, all of which require further investigation. Colchicine is therefore supported by direct and partly replicated randomised controlled trial evidence in cardiac surgery, although the benefit is largely confined to atrial fibrillation rather than mortality or myocardial injury.

##### N-Acetylcysteine

N-acetylcysteine (NAC) has demonstrated antioxidant properties and efficacy in CABG operations, with treated patients showing significantly reduced ROS production in myocardial tissue, decreased TNF-alpha levels in coronary sinus blood, and lower creatine kinase-MB levels at 6 and 12 h postoperatively (61). When incorporated into cardioplegic solutions during on-pump CABG, low-dose NAC effectively reduced myocardial oxidative stress, evidenced by lower malondialdehyde levels and a decreased neutrophil percentage during reperfusion (62). Despite these promising biochemical effects, comprehensive meta-analyses encompassing multiple randomised trials have failed to demonstrate statistically significant improvements in clinically relevant outcomes, including mortality, acute renal failure, atrial fibrillation, or major adverse cardiac events (63, 64), suggesting that biochemical markers may not translate to meaningful clinical benefit. However, subgroup analyses suggest that intravenous NAC may reduce acute kidney injury and arrhythmias, and high-dose regimens may reduce in-hospital mortality (64, 65). This pattern of subgroup benefit within neutral overall trials underscores the need for patient-level stratification to identify those most likely to respond to targeted interventions. In summary, randomised controlled trials of N-acetylcysteine in cardiac surgery have shown overall neutral effects on hard clinical outcomes, with potential benefits emerging only in selected subgroup analyses. Beyond patient heterogeneity, the timing of administration is likely to be a critical determinant of antioxidant efficacy. Because ROS are not uniformly injurious but also act as indispensable signalling intermediates of endogenous cardioprotection, antioxidants given indiscriminately across the perioperative period may suppress the protective redox signalling that underlies ischaemic conditioning at the same time as attenuating the injurious reperfusion burst (12, 13). This consideration applies not only to N-acetylcysteine but to all of the antioxidant strategies discussed in this review, and offers a further explanation for the recurrent discrepancy between encouraging experimental findings and largely neutral clinical results.

##### Corticosteroids

**Figure 4:**
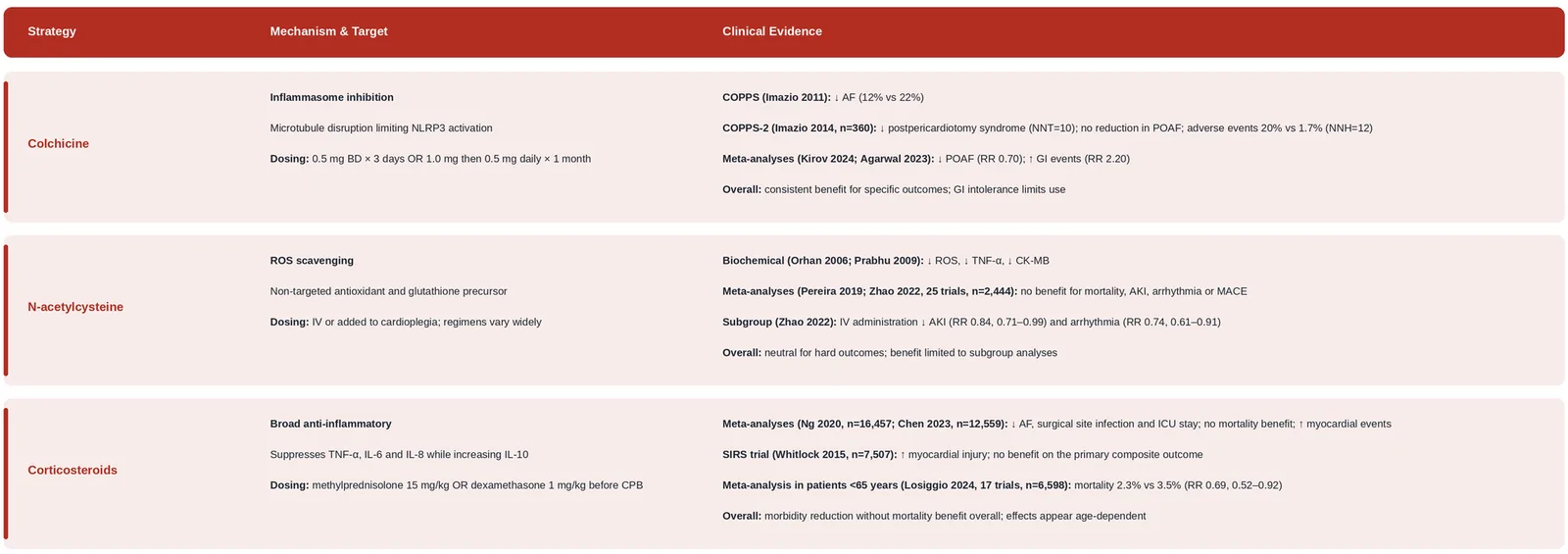
Anti-Inflammatory therapeutic strategies.

Corticosteroids have been shown to attenuate the systemic inflammatory response in cardiac surgery by suppressing pro-inflammatory cytokines. Methylprednisolone at 15 mg/kg administered before CPB in patients undergoing CABG significantly reduced postoperative TNF-alpha, interleukin-6, and interleukin-8 levels whilst increasing anti-inflammatory interleukin-10 release, and was associated with improved cardiac index and oxygen delivery (66). Several meta-analyses of randomised controlled trials including almost 20,000 patients demonstrated a reduction in atrial fibrillation, renal failure, surgical site infection, and intensive care unit length of stay in those receiving perioperative corticosteroids, although there were no differences in in-hospital mortality (67, 68). Importantly, the meta-analysis by Ng and colleagues also identified increased myocardial adverse events with corticosteroid use, and the SIRS trial demonstrated increased myocardial injury without benefit on the primary composite outcome (69). Recent evidence suggests mortality benefit may be age-dependent, with patients under 65 years showing reduced mortality (70). Recent evidence indicates that this mortality effect is age-dependent: a 2024 meta-analysis of 17 randomised trials including 6,598 patients reported a significant reduction in mortality with prophylactic corticosteroids in patients younger than 65 years (risk ratio: 0.69, 95% confidence interval: 0.52–0.92), whereas signals of harm have been observed in older patients (70). The anti-inflammatory agents discussed in this section are compared in Figure 4.

#### Comparative assessment of therapeutic strategies

The agents reviewed above differ substantially not only in mechanism but in the strength and directness of the evidence supporting them, in their safety profiles, and in their proximity to clinical use. Drawing these threads together, Table 1 compares the pharmacological strategies across their mechanistic target, the highest level of evidence available in cardiac surgery, their principal limitations, translational barriers and safety concerns, and their current clinical readiness. Two patterns are apparent. First, the agents with the most direct and replicated cardiac surgical evidence, colchicine and corticosteroids, are not those with the broadest mechanistic reach, and their demonstrable benefits are largely restricted to specific outcomes such as atrial fibrillation. Second, several mechanistically compelling mitochondrial strategies, in particular elamipretide and mitoquinol, remain supported predominantly by preclinical or indirect data, so that mechanistic plausibility and clinical readiness are, at present, poorly aligned. This dissociation reinforces the central argument of this review: that progress depends less on the discovery of new agents than on identifying, in advance, the patients in whom a given mechanism predominates.

**Table 1.** Comparative appraisal of therapeutic strategies for ischaemia-reperfusion injury in cardiac surgery.

Strategy	Mechanistic target	Highest level of evidence in cardiac surgery	Principal limitations	Translational barriers and safety concerns	Clinical readiness
Cyclosporin A	MPTP opening (cyclophilin D)	Randomised trials in cardiac surgery, with benefit confined to subgroups (20, 22)	Neutral for hard clinical outcomes; apparent benefit restricted to patients with longer CPB times and therefore hypothesis-generating	Narrow administration window around aortic unclamping; immunosuppression and nephrotoxicity constrain repeat dosing	Tested but unproven; requires an enriched-population trial
Elamipretide	Cardiolipin and cristae integrity	Ex vivo human myocardium and phase 2 trials in heart failure; no cardiac surgical trial (27, 28)	No cardiac surgical outcome data; existing endpoints are surrogate or imaging-based, and the repeat-dose trial was neutral	Parenteral administration and cost; accelerated approval limited to improving muscle strength in Barth syndrome	Early phase for cardiac surgery
Mitoquinol (MitoQ)	Mitochondria-targeted ROS scavenging	Preclinical models only (31–33)	No human cardiac surgical data; efficacy inferred entirely from animal IRI models	Pharmacologically distinct from oral coenzyme Q10 despite the shared quinone moiety; human perioperative dosing undefined	Preclinical
Coenzyme Q10	Mitochondrial bioenergetics and antioxidant capacity	Several small randomised trials and a meta-analysis of eight trials (34–37)	Small, heterogeneous trials with short-term and largely surrogate endpoints	Requires one to two weeks of preoperative loading, excluding urgent and emergency surgery	Adjunctive use plausible; larger trials required
Doxycycline	MMP-mediated glycocalyx degradation	Single small randomised trial with surrogate endpoints (49)	Small and unreplicated; glycocalyx markers rather than patient-centred outcomes	Three-day preoperative course required; prophylactic antibiotic exposure raises stewardship concerns	Early clinical, unreplicated
Dexmedetomidine	Inflammation-mediated endothelial injury	Single small randomised trial with surrogate endpoints (50)	Surrogate markers only; effect not separable from concurrent sedation and anaesthetic practice	Bradycardia and hypotension; wide variation in perioperative sedation protocols between centres	Early clinical, unreplicated
Operative modification of CPB	Mechanical microvascular stressors	Small mechanistic and observational studies alongside the wider off-pump literature (41)	Confounded by operator experience and patient selection; off-pump surgery is not outcome-equivalent for all patients	Depends on technical expertise and case selection rather than an added therapy; not universally deliverable	Available now within existing practice
Complement inhibition (sCR1/TP10)	Complement cascade and thrombo-inflammation	Randomised trial of 564 patients, neutral for its primary composite endpoint (52, 53)	Benefit in male patients arose from subgroup analysis and was not confirmed in a subsequent trial in female patients	Biologic manufacturing cost; theoretical infection risk from sustained complement blockade	Stalled; requires mechanistic re-evaluation
Recombinant DNase	Neutrophil extracellular traps	Observational human data with experimental model efficacy (54, 55)	No therapeutic trial in cardiac surgery; human data demonstrate association rather than causation	Bleeding risk in the systemically anticoagulated CPB setting is untested	Experimental
Colchicine	NLRP3 inflammasome and microtubule assembly	Multiple randomised trials and meta-analyses (57–60)	Benefit largely confined to atrial fibrillation and postpericardiotomy syndrome; no mortality signal	Gastrointestinal intolerance (RR 2.20) drives discontinuation; narrow therapeutic index in renal impairment	Clinically deployable for specific outcomes
N-acetylcysteine	Non-targeted ROS scavenging	Meta-analyses of randomised trials, neutral overall with a subgroup signal for AKI (63, 64)	Neutral for mortality, atrial fibrillation and acute kidney injury overall; route and dose highly heterogeneous	Indiscriminate perioperative antioxidant exposure may suppress the protective redox signalling underlying conditioning	Not recommended outside trials
Corticosteroids	Broad cytokine suppression	Large randomised trials and meta-analyses, including one trial of 7,507 patients and a meta-analysis of 16,457 patients (67, 69, 70)	Morbidity benefit without mortality benefit, accompanied by a myocardial injury signal; the supporting mortality meta-analysis enrolled only patients younger than 65 years	Hyperglycaemia and infection risk; signals of harm in patients aged 65 years and over	Clinically deployable with age-based caution

Randomised trials are reported at the highest level of evidence available in cardiac surgical populations; where no such evidence exists, the highest available preclinical or non-surgical evidence is stated. AKI, acute kidney injury; CPB, cardiopulmonary bypass; IRI, ischaemia-reperfusion injury; MMP, matrix metalloproteinase; MPTP, mitochondrial permeability transition pore; NET, neutrophil extracellular trap; ROS, reactive oxygen species; RR, risk ratio.

#### Why cardioprotection has failed in clinical translation

The persistent translational gap between mechanistically rational preclinical cardioprotection and consistently neutral or modest clinical trials has now been documented across multiple agents and mechanistic classes (71, 72). Recognising the principal reasons for this pattern is a prerequisite for designing strategies that succeed where their predecessors have not, and is essential context for the multi-omics and artificial intelligence framework introduced in the following sections. First, preclinical efficacy is most often demonstrated in young, healthy animal models subjected to single-vessel occlusion and reperfusion, whereas trial participants are predominantly older patients with hypertension, diabetes, hyperlipidaemia, and established coronary disease, frequently taking multiple cardiovascular medications. Both the comorbidities themselves and concomitant agents such as statins, sulphonylureas, beta-blockers, nitrates, and renin-angiotensin inhibitors interact with the very signalling pathways that cardioprotective interventions seek to engage, attenuating or abolishing their effect (72). This problem of comorbidity- and co-medication-related interference is rarely modelled preclinically and goes some way to explaining why agents that succeed in the laboratory fail at the bedside. Second, most agents tested in cardiac surgery have targeted a single mechanistic node within an injury process that is multi-mechanistic and self-amplifying. Monotherapy directed at mitochondrial permeability transition, oxidative stress, or inflammation alone has consistently failed to produce a robust clinical signal, and there is now broad consensus that effective protection will likely require combination strategies addressing several mechanisms simultaneously (11, 73).

Third, trial design and endpoint selection have themselves limited inference. Many cardiac surgical trials are powered for surrogate biomarkers such as troponin release rather than patient-centred clinical endpoints, while trials powered for hard outcomes are frequently underpowered to detect mechanism-specific benefit in the subgroups in which it is most plausible. Reported subgroup signals, such as those for cyclosporin A in patients with longer cardiopulmonary bypass times and for corticosteroids in patients younger than 65 years, are therefore typically hypothesis-generating rather than confirmatory (22, 70).

Underlying these specific issues is the unaddressed problem of patient heterogeneity. Identical interventions are administered to patients whose mitochondrial reserve, inflammatory tone, and microvascular susceptibility differ substantially, with the consequence that genuine benefit in a responsive subgroup is diluted by neutral or even adverse effects in non-responders. Strategies for prospectively identifying responsive subgroups, the focus of the following sections on multi-omics and artificial intelligence, are therefore not an adjunct to future cardioprotective trial design but a precondition for it.

### Introduction to multi-omics

The therapeutic strategies discussed above represent meaningful advances in targeting specific IRI mechanisms, yet many have shown inconsistent clinical outcomes. The fundamental limitation is not the absence of effective pharmacological targets but rather the inability to identify, prospectively and at the individual level, which patients harbour particular pathophysiological vulnerabilities and which interventions will address them. Multi-omics technologies, integrating genomics, epigenomics, transcriptomics, proteomics, and metabolomics, offer a paradigm shift from uniform cardioprotection towards molecularly informed, individualised strategies (74, 75).

Each layer captures a distinct biological dimension: genomics defines inherited susceptibility; epigenomics reveals dynamic remodelling of gene regulatory programmes in response to surgical stress; transcriptomics captures real-time expression changes during ischaemia and reperfusion; proteomics characterises the functional effectors of these changes; and metabolomics provides an integrated readout of upstream events as they manifest in cellular energetics and metabolic flux (8, 76). Integrated across layers and combined with machine learning, this multimodal data enables a systems-level understanding of IRI grounded in network medicine, which recognises that injury heterogeneity arises from the cumulative, interdependent activity of multiple biological networks that differ between patients, and provides the substrate for patient stratification that population-averaged clinical risk scores cannot replicate (9, 75, 77, 78).

### Genomic and epigenomic profiling

Genome-wide studies have identified polymorphisms in genes encoding inflammatory mediators, adhesion molecules, and stress-response proteins that influence IRI susceptibility (79, 80). Podgoreanu and colleagues demonstrated that polymorphisms in interleukin-6, intercellular adhesion molecule-1, and E-selectin independently predicted postoperative myocardial infarction after CABG, with genotypic information improving predictive models beyond clinical risk factors alone (79). Westphal and colleagues extended this through a genome-wide association study within the RIPHeart trial, identifying variants associated with postoperative myocardial infarction, atrial fibrillation, acute stroke, acute kidney injury, and delirium, demonstrating the breadth of outcomes influenced by germline variation (80). Jouan and colleagues further showed that interleukin-6 and interleukin-10 polymorphisms predicted a composite endpoint of death, low cardiac output syndrome, myocardial infarction, sepsis, and acute renal insufficiency following cardiac surgery, collectively supporting the potential for preoperative genomic screening to identify patients requiring targeted cardioprotection (81).

Epigenetic modifications provide complementary, dynamic regulatory mechanisms. Fischer and colleagues identified DNA methylation changes within 24 h of cardiac surgery associated with new-onset atrial fibrillation (82). Epigenomic profiling thus captures the patient's temporal molecular state, including the cumulative impact of comorbidities and prior ischaemic burden, rather than simply inherited susceptibility. MicroRNAs add a further regulatory dimension: ischaemic preconditioning upregulates miR-21, which protects cardiomyocytes against IRI by suppressing the pro-apoptotic gene PDCD4 (83), whilst circulating miR-499 peaks 1–3 h post-surgery, significantly earlier than troponin I, with superior diagnostic performance for perioperative myocardial injury (84). The earlier kinetics of miR-499 may enable earlier detection of significant myocardial damage and inform individualised postoperative management.

### Transcriptomic and proteomic insights

Transcriptomic profiling of myocardial biopsies during cardiac surgery reveals substantial changes in genes regulating inflammatory signalling, oxidative stress, and cellular survival (85). Critically, transcriptomic profiles varied substantially between patients despite standardised surgical and cardioplegic conditions, confirming that individual molecular responses to ischaemia cannot be inferred from operative characteristics alone. Patients with suppressed mitochondrial biogenesis genes or elevated nuclear factor kappa-B pathway activity represent distinct risk phenotypes that map directly onto the pharmacological targets discussed above, making them the most plausible candidates for prophylactic intervention with elamipretide, colchicine, or coenzyme Q10.

Similarly, proteomic biomarkers can provide clinically useful prognostic information beyond standard risk scores. Several individual proteins have demonstrated predictive value: preoperative levels of vascular cell adhesion molecule-1, a marker of endothelial inflammation, predict late mortality after CABG (86), whilst high mobility group box 1 measured after cardiopulmonary bypass independently predicts a composite of serious complications including death, myocardial infarction, stroke, renal failure, and prolonged ventilation, implicating innate immune activation as a potential therapeutic target (87). Because IRI involves simultaneous disruption across multiple biological pathways, no single protein can adequately characterise the full extent of injury. Multi-marker panels therefore outperform individual biomarkers: combinations of soluble ST2, galectin-3, and NT-proBNP, each reflecting a different aspect of cardiac stress and injury, predict cardiovascular events and mortality following surgery (88), and a simple score incorporating age, soluble urokinase plasminogen activator receptor, and NT-proBNP outperforms EuroSCORE II for predicting intermediate and long-term mortality (89). Combining proteomic data with transcriptomic profiling strengthens this further, as concordant abnormalities identified at both the gene expression and protein level provide greater confidence in biological relevance and reduce the noise inherent to either platform used alone (8, 76).

### Metabolomic signatures

Metabolomics reflects the integrated functional output of all upstream molecular events as they are expressed in real time through altered metabolic flux, and occupies a translational position within the multi-omics hierarchy (74). Chouchani and colleagues demonstrated that succinate selectively accumulates in ischaemic myocardium and, upon reperfusion, is rapidly oxidised, driving mitochondrial ROS production through reverse electron transport at complex I (90). This mechanism directly links the metabolic signature of ischaemia to the oxidative burst of reperfusion, illustrating how metabolomic profiling can expose mechanistic targets not detectable by conventional biomarker approaches.

Clinically, Maltesen and colleagues monitored 57 metabolites longitudinally across the surgical ischaemia-reperfusion sequence and identified distinct temporal patterns of metabolic disruption with marked inter-patient variability despite comparable operative conditions, demonstrating the feasibility of pre-ischaemic metabolic profiling for prospective risk identification (91). Turer and colleagues showed that patients with pre-existing left ventricular dysfunction exhibit a qualitatively distinct metabolic response to reperfusion, characterised by global suppression of fuel uptake and critically limited metabolic reserve, correlating with prolonged inotropic requirements (92). This phenotype of metabolic vulnerability is not detectable by standard haemodynamic assessment and could prospectively identify patients requiring tailored cardioplegia or perioperative metabolic support.

### Multi-Omics integration and the path to personalised cardioprotection

The clinical utility of multi-omics lies in integration across layers, which enables identification of coherent molecular phenotypes with far greater fidelity than any single assay (75, 76). Integrated profiling could classify patients into molecularly distinct subgroups before surgery: those with mitochondrial vulnerability, heightened inflammatory activation, microvascular fragility, or metabolic decompensation, each requiring a different cardioprotective strategy.

This approach also offers a methodology for resolving the consistent failure of mechanistically rational agents in population-level trials. *Post hoc* analyses of the cyclosporin A CABG trial suggested benefit in patients with longer CPB times, indicating a responsive molecular subgroup (21, 22). Prospective stratification by mitochondrial stress signatures or metabolomic markers of mitochondrial permeability transition pore sensitivity could have identified this subgroup and avoided dilution by non-responders, transforming a neutral trial result into actionable evidence (8, 73). Similarly, Kornej and colleagues reviewed how integrated multi-omics approaches may improve atrial fibrillation risk prediction (93), suggesting that preoperative molecular profiling could enable targeted prophylaxis in the substantial proportion of patients who develop postoperative atrial fibrillation, reducing both complication rates and indiscriminate pharmacological exposure.

### Artificial intelligence: bridging molecular evidence and clinical outcomes

A fundamental challenge in translating IRI research to clinical practice is the disconnect between biochemical evidence of myocardial injury and clinically meaningful outcomes. As discussed, patients demonstrate substantial heterogeneity in IRI manifestations despite similar operative characteristics and biomarker elevations. Network medicine addresses this heterogeneity by moving beyond linear cause-effect models to comprehensively map molecular interaction patterns (77). Instead of identifying single risk variants, this approach examines how genetic predisposition, protein expression, and metabolic states collectively interact to determine event susceptibility (75). This reflects the clinical reality that IRI diversity arises not from single molecular defects but from the cumulative effects of multiple interconnected pathways operating differently in each individual.

Translating this molecular complexity into clinical utility requires computational approaches capable of handling large multidimensional datasets. Artificial intelligence and machine learning provide this capability, offering the analytical infrastructure to construct patient-specific predictions from complex molecular networks whilst continuously refining output with new data (94, 95). Unlike traditional statistical models that assume linear relationships between individual biomarkers and outcomes, artificial intelligence can identify complex, non-linear interactions across multiple biological layers. For instance, a patient's genetic predisposition to mitochondrial dysfunction may only manifest as clinically significant IRI in the context of specific inflammatory profiles, metabolic states, and surgical stressors, patterns that emerge only through integrated analysis of genomic, proteomic, and metabolomic data. Meta-analyses confirm that machine learning models provide significantly better discrimination in mortality prediction after cardiac surgery compared with traditional logistic regression (96).

Early applications demonstrate the capacity of artificial intelligence to predict cardiovascular outcomes and guide interventions (97, 98). In the context of myocardial protection, artificial intelligence models trained on preoperative multi-omics profiles could stratify patients into distinct risk phenotypes before surgery. Patients identified with mitochondrial vulnerability signatures could receive prophylactic elamipretide or an antioxidant like Coenzyme Q10, whilst those with inflammatory activation profiles might benefit from colchicine or targeted corticosteroids. Similarly, patients with microvascular dysfunction markers could receive enhanced glycocalyx protection strategies. Beyond outcome prediction, AI-driven drug-target interaction modelling, spanning machine-learning, deep-learning, network-based, and hybrid multi-omics approaches, offers a complementary route to patient-specific therapy selection and to the repurposing of existing agents for cardioprotection (99).

**Figure 5:**
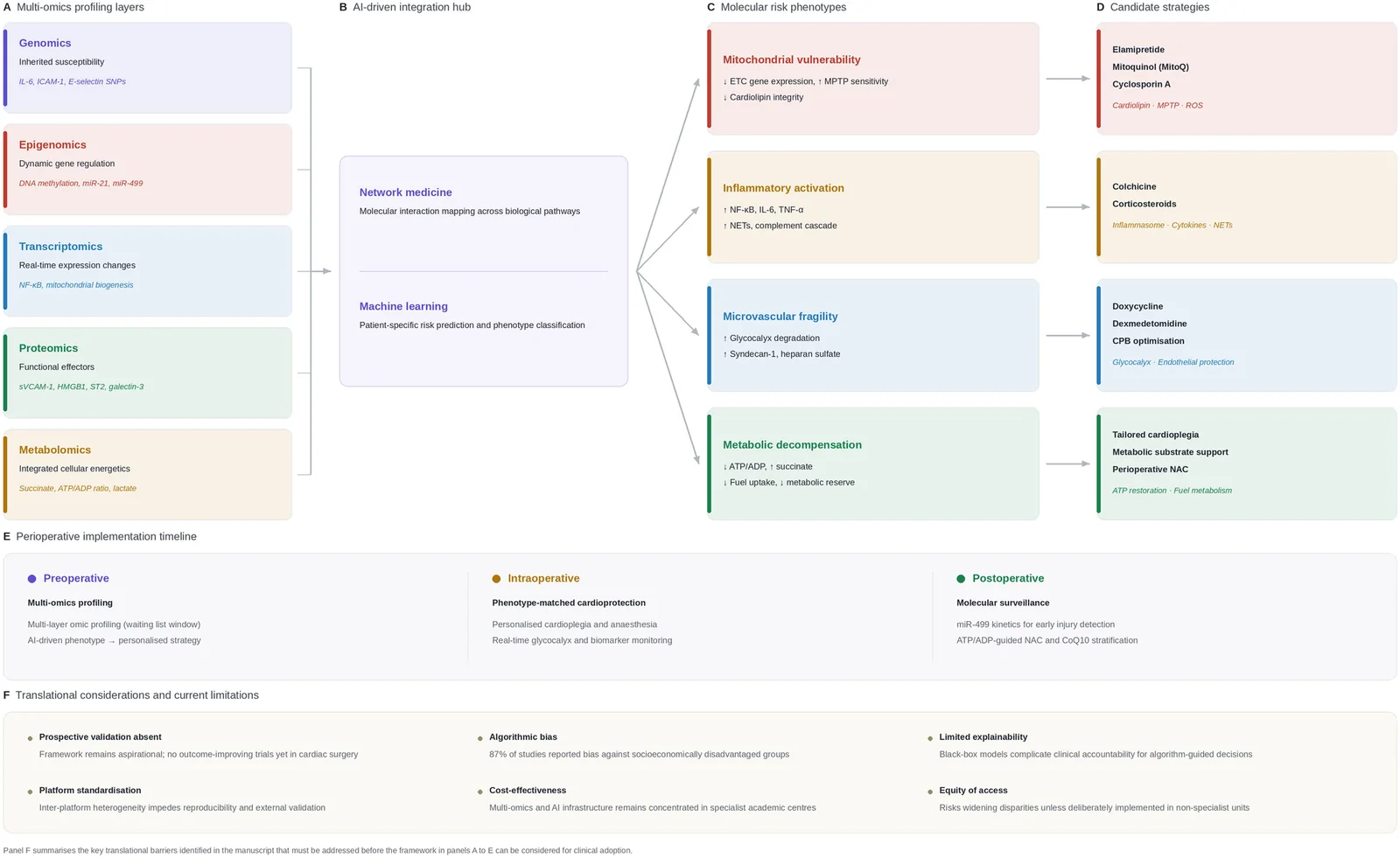
Proposed framework for multi-omics integration for personalised cardioprotection in cardiac surgery. **(A)** Five omics layers: genomics, epigenomics, transcriptomics, proteomics, and metabolomics, with representative molecular markers of ischaemia-reperfusion injury susceptibility identified in the cardiac surgical literature. **(B)** AI-driven integration hub combining network medicine for molecular interaction mapping across biological pathways with machine learning for patient-specific risk prediction and phenotype classification. **(C)** Four molecularly defined risk phenotypes stratified by predominant injury mechanism: mitochondrial vulnerability, inflammatory activation, microvascular fragility, and metabolic decompensation. **(D)** Example mechanism-matched candidate strategies for each phenotype, presented irrespective of current evidence strength. **(E)** Perioperative implementation timeline illustrating the concept of preoperative multi-layer omic profiling during the surgical waiting list window with AI-driven phenotype assignment, intraoperative allocation of personalised cardioplegia, anaesthesia, and real-time biomarker monitoring, and postoperative molecular surveillance including circulating biomarkers such as miR-499 kinetics for early injury detection and ATP/ADP-guided stratification for N-acetylcysteine and coenzyme Q10. **(F)** Translational considerations and current limitations, summarising the key barriers, including absent prospective validation, algorithmic bias, limited explainability, platform standardisation, cost-effectiveness, and equity of access, that must be addressed before the framework in panels A to E can be considered for clinical adoption.

This preoperative risk stratification enables mechanism-informed therapy selection, moving beyond the current uniform approach to truly personalised cardioprotection protocols. By integrating the conceptual framework of network medicine with the computational power of machine learning, multi-omics profiling can be developed into actionable clinical decision support tools for personalised myocardial protection. This proposed framework is illustrated in Figure 5.

It must be emphasised, however, that this framework remains aspirational rather than established. Although multi-omics platforms and machine-learning methods already exist, their integration into a validated, outcome-improving clinical pathway for myocardial protection has not been achieved. Realising this vision will require adequately powered prospective studies demonstrating that omics-guided, AI-informed cardioprotection improves patient-centred outcomes rather than surrogate markers. At present, therefore, the integration of multi-omics with artificial intelligence is best regarded as a promising direction for research, with the potential to help explain previous inconsistent trial results, rather than as a clinically ready strategy.

## Discussion

The preoperative pathway offers the highest yield for constructing multi-omics-driven molecular profiles to enable risk stratification and optimisation of cardioprotective strategies. Given current cardiac surgical waiting lists, this window provides time for comprehensive molecular profiling. Potential applications include preoperative omic characterisation of adrenergic receptor sensitivity and inflammatory pathways to enable personalised anaesthesia selection and pre-emptive immunomodulation strategies including colchicine therapy (57, 100).

Postoperative multi-omics surveillance could follow as the second implementation stage, providing a controlled environment to validate predictive models before deployment in time-critical intraoperative settings. Early postoperative molecular profiling could enable targeted therapeutic interventions. For instance, proteomic and metabolomic evidence of ongoing mitochondrial dysfunction, characterised by elevated markers of lipid peroxidation and decreased ATP to ADP ratios, could stratify patients for coenzyme Q10 supplementation or N-acetylcysteine administration (34, 37). Across both phases, multi-omics profiling of microvascular dysfunction could enable clinically impactful therapeutic decision-making. During surgery, proteomic detection of endothelial glycocalyx degradation markers could guide real-time adjustments to CPB protocols (39, 48).

### Technical capabilities and clinical gaps

Whilst multi-omics and artificial intelligence demonstrate considerable promise in research environments, their clinical translation remains constrained by fundamental limitations. Current studies are predominantly retrospective, and prospective validation is notably absent from cardiac surgical practice. Robust evidence demonstrating improved patient outcomes through artificial intelligence-driven multi-omics guided strategies remains to be established.

Logistical barriers compound these challenges. Multi-omics technologies require specialised equipment, extensive technical expertise, and sophisticated bioinformatic infrastructures (76). Processing turnaround times remain incompatible with current surgical decision-making windows, though recent advances in rapid multi-omics have achieved turnaround times of two to three days for genomic sequencing in acute care settings, suggesting preoperative profiling may be feasible given typical cardiac surgery waiting times (101). Platform heterogeneity introduces analytical challenges, as different systems may generate inconsistent results for identical samples, complicating development of standardised analytical protocols (102). Financial implications represent an additional barrier to widespread adoption (78).

### Challenges of artificial intelligence

Machine learning and artificial intelligence offer powerful tools for analysing complex multi-omics data, but clinical translation presents particular challenges. A fundamental limitation is the opaque nature of many artificial intelligence systems, in which predictions are generated through complex computational processes that clinicians may not easily interpret or explain to patients (103, 104). This raises important questions about clinical accountability when algorithm-guided decisions lead to unfavourable outcomes.

Another significant concern is whether models can be reliably applied across different patient populations. Models developed using data from specific groups may not perform equally well in patients with different characteristics, leading to biased outputs (105). Most published algorithms are validated only on limited datasets from single institutions and most machine learning algorithms evaluated for bias demonstrate bias on sociodemographic factors, with 87% of studies reporting bias against groups with socioeconomic disadvantage (106). Healthcare institutions require substantial infrastructure and technical resources that may not be available in many settings, potentially worsening disparities where personalised medicine approaches remain accessible primarily to well-resourced academic centres.

Taken together, these limitations indicate that the translational distance between current capability and routine clinical use remains considerable.

## Conclusion

Despite significant advances in cardiac surgical techniques and perioperative care, myocardial injury remains a substantial contributor to morbidity and mortality. The persistent translational gap between mechanistically informed pharmacological interventions and clinical efficacy reflects a fundamental limitation: the application of uniform therapeutic strategies to a pathophysiologically heterogeneous patient population.

The convergence of multi-omics technologies and artificial intelligence offers a potential pathway beyond this impasse, enabling comprehensive molecular characterisation of individual patients and stratification for targeted interventions matched to personalised pathophysiological mechanisms. Despite considerable potential, significant challenges persist in developing reproducible artificial intelligence-driven omics algorithms that demonstrably improve patient outcomes, exhibit cost-effectiveness, and can be successfully implemented within existing healthcare infrastructure.

## Future directions

Several key research priorities warrant investigation. A fundamental challenge is the systematic characterisation of molecular phenotypes that predict individual susceptibility to IRI. Large-scale prospective studies integrating baseline omic profiling with longitudinal clinical outcomes are essential to identify molecular signatures that stratify patients into distinct risk categories and establish comprehensive temporal maps of myocardial injury and recovery. Beyond immediate clinical applications, multi-omics and artificial intelligence offer novel capabilities for clinical trial methodology by utilising omic profiles to guide patient selection and assess drug efficacy. Grouping patients according to molecular profiles may provide more robust trial results, leading to the development of pharmacological protocols for distinct cardiac surgical patient groups.

The logistical challenges of implementing multi-omics and artificial intelligence technology will require substantial investment in infrastructure development, methodological standardisation, and ethical governance frameworks. Multi-institutional research networks must establish standardised protocols encompassing sample collection, processing, and analytical procedures. Cost-effectiveness analyses and equitable access strategies are fundamental to preventing widening healthcare disparities.

Successfully addressing these challenges requires sustained interdisciplinary collaboration among cardiac surgeons, basic scientists, bioinformaticians, data scientists, ethicists, and policymakers. The next decade will determine whether multi-omics and artificial intelligence fulfil their promise to transform cardiac surgery. The biological potential is clear, the technological capabilities are advancing rapidly, and the clinical need is undeniable. What remains is the difficult work of rigorous clinical validation, addressing implementation barriers, ensuring equitable access, and translating scientific promise into improved outcomes for the hundreds of thousands of patients undergoing cardiac surgery annually.
